# Changes in the nonlinear and viscoelastic mechanical properties of the rat lamina cribrosa under chronic high intraocular pressure

**DOI:** 10.3389/fbioe.2026.1875609

**Published:** 2026-08-31

**Authors:** Yuqing Wang, Liu Liu, Jingxi Zhang, Yushu Liu, Jifeng Ren, Xiuqing Qian

**Affiliations:** 1 School of Biomedical Engineering, Capital Medical University, Beijing, China; 2 Department of Medical Engineering, Peking University Third Hospital, Beijing, China; 3 Beijing Friendship Hospital, Capital Medical University, Beijing, China; 4 School of Special Education and Rehabilitation, Shandong Medical And Pharmaceutical University, Yantai, Shandong, China; 5 Beijing Key Laboratory of Intelligent Diagnosis Technology and Equipment for Optic Nerve-Related Eye Diseases, Capital Medical University, Beijing, China; 6 Beijing Key Laboratory of Clinical Engineering Solutions for Mental Health, Capital Medical University, Beijing, China

**Keywords:** high intraocular pressure, lamina cribrosa, indentation, nonlinear, viscoelasticity

## Abstract

**Introduction:**

The lamina cribrosa (LC) in the optic nerve head is the initial site of glaucomatous optic nerve injury. Under high intraocular pressure (IOP), the deformation of the LC compresses the retinal ganglion cell (RGC) axons passing through it. The influence of changes in the nonlinear and viscoelastic properties of the LC on its deformation cannot be neglected under high IOP. Therefore, investigating the changes in the nonlinear and viscoelastic mechanical properties of the LC under different durations of high IOP is of great significance.

**Methods:**

A rat model with chronic high IOP was established by cauterizing the superior scleral vein and injecting 5-fluorouracil (5-FU) under the conjunctiva, producing sustained IOP elevation for 4, 8, and 12 weeks. Indentation tests were conducted on the rat LC. Then the loading segment and stress relaxation segment of the indentation curve were fitted separately.

**Results:**

Compared with the control group, both the nonlinear parameters and viscoelastic parameters of the LC in experimental eyes decreased significantly. In contralateral control eyes, modest changes were also observed in some mechanical parameters, including the nonlinear parameters, relaxation modulus, and instantaneous modulus.

**Discussion:**

The results indicated that long-term elevated IOP may contribute to the progression of glaucomatous optic nerve injury by altering the strain-dependent and time-dependent mechanical responses of the LC.

## 1 Introduction

Glaucoma is a major cause of irreversible blindness. Progressive optic nerve damage (Resnikoff et al., 2004; Susanna et al., 2015) is the main feature of glaucoma, where pathological elevation of intraocular pressure (IOP) is one of its most important risk factors (Pang and Clark, 2007). Accumulating evidence indicates that lamina cribrosa (LC) within optic nerve head (ONH) is the primary site of glaucomatous optic nerve injury (Quigley et al., 1981; Goldberg et al., 2002; Downs and Girkin, 2017). LC deformation induced by sustained high IOP leads to impaired axoplasmic transport and compromised microcirculation, ultimately driving glaucomatous optic neuropathy (Lu et al., 2025; Yan et al., 1994). LC deformation is closely associated with its mechanical properties (Sigal et al., 2011; Coudrillier et al., 2016). As a composite of collagen fibrils and other solid matrix components, LC exhibits complex nonlinear and viscoelastic mechanical behavior (Albon et al., 2000; Li and Song, 2020; Voorhees et al., 2017; Grytz et al., 2012; Karimi et al., 2025; Safa, Read, and Ethier, 2021). Therefore, it is important to investigate how these properties change with the duration of elevated IOP.

Currently, researchers have developed various experimental methods to characterize the mechanical properties of LC, including tensile, compression, indentation, ultrasound elastography, etc. (Spoerl, Boehm, and Pillunat, 2005; Boazak et al., 2019; Braunsmann et al., 2012; Ma et al., 2025; Liu et al., 2022). However, most studies have characterized mechanical behaviors of LC under the assumption of linear elasticity (Du et al., 2019; Braunsmann et al., 2012; Ma et al., 2025; Qian et al., 2021; Sigal et al., 2014; Liu et al., 2022). As a critical load-bearing structure within ONH, LC is composed of a complex porous network and undergoes substantial deformation under elevated IOP, suggesting that it may likewise exhibit significant nonlinear mechanical behavior (Karimi et al., 2021; Downs and Girkin, 2017; Midgett et al., 2017). While the stress–strain curves of LC strips in tensile testing showed typical nonlinearity, a quantitative analysis of the associated mechanical parameters is lacking (Spoerl, Boehm, and Pillunat, 2005). Furthermore, although techniques like acoustic radiation force optical coherence elastography (ARF-OCE) have mapped elasticity variations in the optic nerve head under different pressures, they lack the resolution to specifically isolate the LC from surrounding tissues (Shi et al., 2023). Given the small scale of LC, we employed indentation test to quantitatively characterize its nonlinear mechanical properties.

Meanwhile, LC also exhibits pronounced time-dependent behaviors under sustained or dynamic loading, suggesting that its viscoelastic characteristics may govern the accumulation and recovery of tissue deformation (Zhao et al., 2014; Safa, Read, and Ethier, 2021). Previous studies have demonstrated that the peripapillary sclera shows marked stress relaxation and creep behavior under tensile loading, indicating that its viscoelastic properties play an important role in regulating the local mechanical environment of the ONH (Downs et al., 2005; Jia et al., 2022). Further experimental evidence has confirmed that posterior ocular tissues exhibit significant time-dependent mechanical responses under tensile loading, which can be adequately described using linear viscoelastic models (Song et al., 2023). By combining micromechanical testing of porcine ONH tissues with finite element modeling, Safa et al. explained the time-dependent behavior as a combination of fluid-dependent mechanisms and solid-matrix viscoelasticity (Safa, Read, and Ethier, 2021). Our previous study demonstrated the viscoelastic behavior of the cat LC by measuring changes in LC thickness at different durations of elevated IOP using optical coherence tomography (OCT) (Zhao et al., 2014). However, direct experimental investigations into the viscoelastic mechanical properties of the LC remain limited, and the manner in which these properties evolve with the duration of elevated IOP is still unclear. Therefore, a systematic investigation of viscoelastic behaviors of LC tissues is necessary to more accurately characterize their biomechanical responses under dynamic IOP.

The structure of LC is progressively remodeled under conditions of chronic high IOP (Strickland et al., 2022; Zhang et al., 2022). The changes of LC microstructure will result in the change of mechanical properties. Compared to normal controls, the Young’s modulus of LC decreased by over 40% in Pseudoexfoliation glaucoma (Braunsmann et al., 2012). Our study also showed that the elastic moduli of the glial LC and RGC axons in rats varied with the duration of elevated IOP (Ma et al., 2025). Therefore, in the present study, a chronic high IOP rat model was established by cauterizing the episcleral veins in combination with subconjunctival injection of 5-fluorouracil (5-FU). Indentation experiments were performed on LC region tissue sections to obtain the nonlinear and viscoelastic mechanical parameters of the LC tissues under sustained elevated IOP. In addition, nonlinear mechanical parameters were measured on longitudinal cryosections of the LC and compared with those obtained from transverse sections to examine the anisotropy of the nonlinear mechanical behavior of the LC tissues. This study provides additional biomechanical insight into optic nerve injury and may contribute to a better understanding of glaucoma.

## 2 Materials and methods

### 2.1 Establishment of the high IOP animal model

The animal experiments were conducted in accordance with the principles for the treatment of animals as described in the Statement for the Use of Animals in Ophthalmic and Visual Research by the Association for Research in Vision and Ophthalmology (ARVO). A total of 28 male adult Sprague-Dawley rats (270–300 g) were used in this study, provided by the Laboratory Animal Center of Capital Medical University. The animals were divided into a blank control group (n = 10) and experimental groups with sustained elevated IOP for 4, 8, and 12 weeks (n = 6).

A chronic high IOP model in rats was established based on relevant literature (Zhang et al., 2022; Ma et al., 2025). The rats were anesthetized by intraperitoneal injection of 1% sodium pentobarbital in physiological saline at a dose of 0.4 mL/100 g body weight. Corneal surface anesthesia of the right eye was then performed using oxybuprocaine hydrochloride eye drops (Santen Pharmaceutical, Osaka, Japan). The right eye was chosen as the experimental eye and treated with a high-temperature cautery pen to cauterize 3–4 main episcleral veins. Following the model establishment, subconjunctival injection of 5-FU (Shanghai Xudong Haipu Pharmaceutical Co., Ltd., Shanghai, China) was administered in the experimental eyes to inhibit neovascularization, and ofloxacin eye drops were applied to prevent inflammation. The left eye served as the contralateral control. Samples in this condition are referred to as the contralateral control group. At the end of the experiment, all rats were euthanized by gradual-fill CO_2_ inhalation at a displacement rate of 30% of the chamber volume per minute. Death was confirmed by the cessation of respiration and the absence of response to painful stimuli.

IOP measurements were performed using a Tonolab tonometer (iCare, Vantaa, Finland) every 3 days for both eyes. If the IOP in the right eye exceeded 25 mmHg, 5-FU injection was continued; if the IOP dropped below 25 mmHg and significant neovascularization was observed, cauterization and 5-FU injection were repeated. These procedures were continued until the elevated IOP was maintained for 4, 8, and 12 weeks, respectively.

### 2.2 Collection and processing of LC tissue

The rat eyeballs were enucleated, retaining approximately 2 mm of the optic nerve and removing the anterior segment tissue and excess tissue around the ONH, while preserving the optic nerve and ONH. The tissue was embedded in OCT (Sakura Finetek United States, Torrance, CA, United States) compound along the ONH and then frozen in liquid nitrogen. The study included 10 left-eye samples from the blank control group, along with 6 right-eye samples from the experimental groups (4W, 8W, 12W) and their corresponding contralateral control groups (left-eye samples). These samples were used to prepare cross-sectional sections of the LC region tissue. Additionally, 10 right-eye samples from the blank control group were prepared for longitudinal sections along the optic nerve to study the anisotropy of the mechanical properties of the LC. The frozen sections were cut to a thickness of 50 μm, and neither fixed nor dehydrated, and stored at −20 °C. Prior to indentation testing, the tissues were allowed to warm for 15–20 min, washed 4 times with PBS buffer, each wash lasting 5 min, to remove the OCT embedding medium.

### 2.3 Indentation testing

**FIGURE 1:**
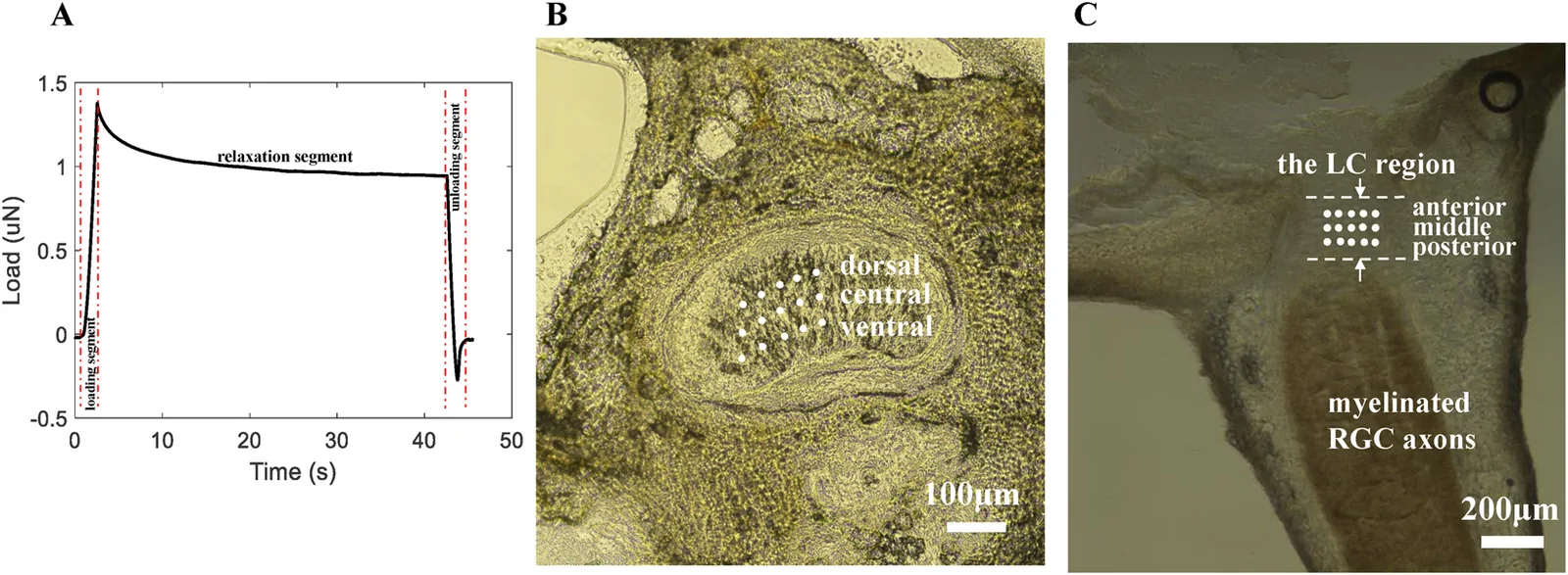
**(A)** Force-time curve obtained from the indentation experiment. The loading segment, relaxation segment, and unloading segment are marked with red dashed lines. **(B)** Schematic diagram of cross-sectional sections of the LC region tissue under the microscope view of the indentation device. Indentations were performed along straight lines at the ventral, central, and dorsal regions, with an approximately 40 μm interval between each indentation. **(C)** Schematic diagram of longitudinal section of the LC region tissue under the microscope view of the indentation device. Five indentations were performed along a straight line at the anterior, middle, and posterior regions, with an approximately 40 μm interval between each indentation.

Indentation tests were performed using the Piuma nanoindenter (Piuma Chiaro, OPTICS 11, Netherlands). Considering that the elastic modulus of the rat LC obtained by AFM is approximately 300 kPa (Liu et al., 2022; Ma et al., 2025), and that the *in vivo* shear modulus of the human lamina cribrosa is approximately 73.1 kPa (Zhang et al., 2020), the cantilever stiffness of the indenter was selected as 0.5 N/m (Skelton et al., 2024). A spherical probe with a radius of 29.5 μm was chosen. The probe parameters, including the cantilever spring constant and probe radius, were checked to ensure consistency with the manufacturer-provided values. The probe was pre-wetted and immersed in the testing solution, followed by optical calibration of the OP1550 interferometer. After the automatic surface-finding procedure, the probe was brought into contact with a blank glass surface used as a rigid reference surface, and the “Geo Factor in air” was calibrated. The calibrated value was compared with the factory-set value of the probe, and only probes with a calibrated-to-factory Geo Factor ratio greater than 0.7 were used for subsequent indentation experiments. Before matrix scanning, a preliminary single-point indentation was performed to confirm appropriate surface detection and reasonable indentation curves. The overall experimental workflow, including tissue collection, embedding orientation, cryosectioning, indentation testing, and mechanical analysis, is summarized in Supplementary Figure S1. The indentation process is as follows: (1) The probe is loaded from the origin at a speed of 5 μm/s until the indentation depth reaches approximately 5 μm, and the loading segment of the indentation curve is obtained; (2) The probe is held at an indentation depth of about 5 μm for 40 s to obtain the stress relaxation segment of the indentation curve; (3) The probe is unloaded at a speed of 5 μm/s back to the origin to obtain the unloading segment of the indentation curve. A complete force-time curve is obtained for each indentation (Figure 1A), including the loading segment, stress relaxation segment, and unloading segment (marked by red dashed lines in Figure 1A). A hold time of 40 s was selected to capture the major part of the stress-relaxation response while minimizing experimental drift (Yoo et al., 2011; Wahlquist et al., 2017; Darling et al., 2009).

To study the regional differences in the LC, indentations were performed at five points along each of three parallel reference lines in three different regions of each sample transverse section (Figure 1B), with approximately 40 μm spacing between each indentation point.

From the longitudinal section of the LC region tissue under the inverted microscope view of the indentation device (Figure 1C), it can be observed that the RGC axons within the LC are unmyelinated and have higher transparency, while the pure RGC axons in the posterior LC are myelinated and appear as opaque, darker areas under the microscope. This characteristic was used to determine the boundary of the posterior LC, as indicated by the white dashed lines on the left side (Figure 1C). The area between the two white dashed lines represents the LC. For each sample slice, five indentations were performed along a straight line at the anterior, middle, and posterior regions, with an approximately 40 μm interval between each indentation point.

### 2.4 Determination of nonlinear parameters of the LC

Spherical indentation generates a complex localized three-dimensional stress field. When a material indented by a spherical indenter is approximated as a semi-infinite half-space, the Hertz equation can be used to describe the linear relationship between the applied force and the resulting indentation depth (Johnson, 1985). For soft materials, the relationship of stress and strain is nonlinear, and Neo-Hookean, Mooney-Rivlin, and polynomial mathematical are used to describe the material behavior (Lin et al., 2009). The LC could be approximated as a homogeneous, nonlinear, and nearly incompressible material. We chose Neo-Hookean model to describe the nonlinear behavior of LC. This model be represented as

$$W=C_{10}\left(\bar{I_{1}}-3\right)+{\frac{1}{D_{1}}\left(J-1\right)}^{2}$$ (1)

where *W* is strain energy density, *C*_10_ is nonlinear parameter, $\bar{I_{1}}$ is isochoric first invariant, *D*_1_ is bulk compressibility parameter, *J* is volume deformation ratio. For incompressible material, the second term in the above equation is zero and can be neglected.

For neo-Hookean materials, following the approach of Huang (Huang, Camras, and Yuan, 2015), the nonlinear parameter *C*_10_ was obtained by fitting Equation 2 to the load–indentation depth data from the loading segment of the indentation curve (Figure 1A).

$$F=\frac{8C_{10}}{\left(1-v^{2}\right)}R^{2}\beta^{\frac{3}{2}}\left(1+\alpha \beta^{\frac{1}{2}}\right)$$ (2)

where F is the indentation load of the probe on the sample, v is the Poisson’s ratio, which is taken as 0.5 for incompressible materials, R is the radius of the spherical probe, β = *h*/ R , where *h* is the indentation depth. In this case, it must satisfy *β* ≤ 0.5, *α* = − 0.089 (Huang, Camras, and Yuan, 2015; Huang et al., 2016).

### 2.5 Determination of viscoelastic parameters of the lamina cribrosa

Monitoring the stress-relaxation response of a sample under constant deformation is a common method for characterizing viscoelasticity. Accordingly, the viscoelastic parameters were obtained by fitting the stress-relaxation segment of the indentation load–time curve (Figure 1A). Some studies discovered the viscoelastic stress-strain relationship for indentation of isotropic incompressible materials with a rigid probe. For Neo-Hookean materials, we referred to Darling et al.'s derivation method and used the nonlinear empirical Formula to describe the viscoelastic stress-strain relationship of LC, as shown in Equation 3 (Darling, Zauscher, and Guilak, 2006):

$$F=\frac{4E_{r}}{3\left(1-v\right)}R^{2}\beta^{\frac{3}{2}}\left(1+\alpha \beta^{\frac{1}{2}}\right)\left(1+\frac{t_{\sigma }-t_{\varepsilon }}{t_{\varepsilon }}e^{-\frac{t}{t_{\varepsilon }}}\right)$$ (3)

By fitting Equation 3 to the stress-relaxation segment of the indentation load–time curve, the relaxation modulus *E_r_*, the relaxation time *t*_σ_ under constant load, and the relaxation time *tε* under constant deformation were obtained as fitting parameters. The instantaneous modulus *E*_0_ and apparent viscosity μ were then calculated using Equations 4 and 5, respectively.

$$E_{0}=E_{r}\left(1+\frac{t_{\sigma }-t_{\varepsilon }}{t_{\varepsilon }}\right)$$ (4)

and

$$\mu =E_{r}\left(t_{\sigma }-t_{\varepsilon }\right)$$ (5)

### 2.6 Statistical analyses

A one-way analysis of variance (ANOVA) was performed to compare data among multiple groups, with statistical significance set at *P* < 0.05. Post hoc pairwise comparisons were carried out using the least significant difference (LSD) test, with *P* < 0.05 considered statistically significant. Spearman correlation analysis was conducted to assess the relationship between the nonlinear and viscoelastic parameters and the duration of elevated IOP using SPSS 26.0 (IBM Corporation, Armonk, United States), with *P* < 0.05 indicating statistical significance. Independent sample t-tests were used to compare differences between cross-sectional and longitudinal sections, with *P* < 0.05 considered statistically significant.

## 3 Results

### 3.1 IOP measurement

**FIGURE 2:**
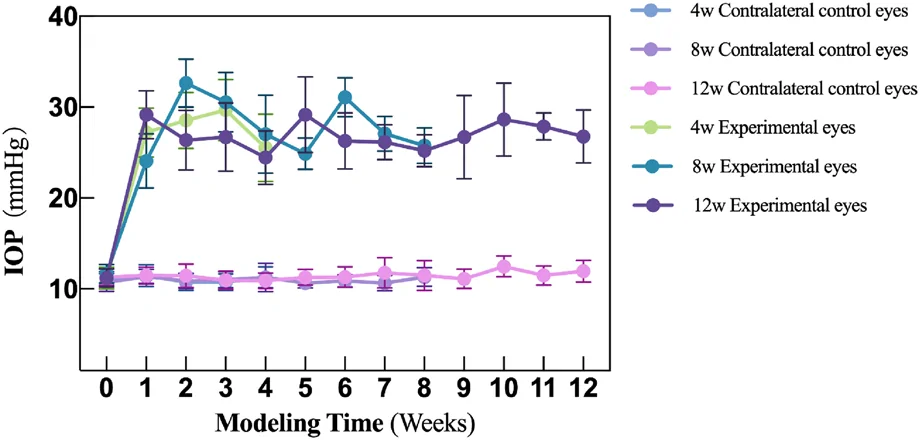
IOPs of both eyes in experimental groups (n = 6). The IOPs of experimental eyes increased significantly after model induction (*P* < 0.05) and the IOPs of contralateral control eyes remained at normal level (*P* > 0.05). Error bars represent the standard deviation (SD).

Before experimentation, IOP of all rats was measured and fell within the normal range. The high IOP group rats underwent weekly IOP measurements after the experimental procedure until euthanasia. Following model induction, IOP of experimental eyes significantly increased (Figure 2) and remained above 25 mmHg. IOP of contralateral control eyes showed no significant changes throughout the experiment, indicating that the sustained pathological high IOP in experimental eyes did not significantly affect the IOP of contralateral control eyes.

### 3.2 Nonlinear properties of LC

**FIGURE 3:**
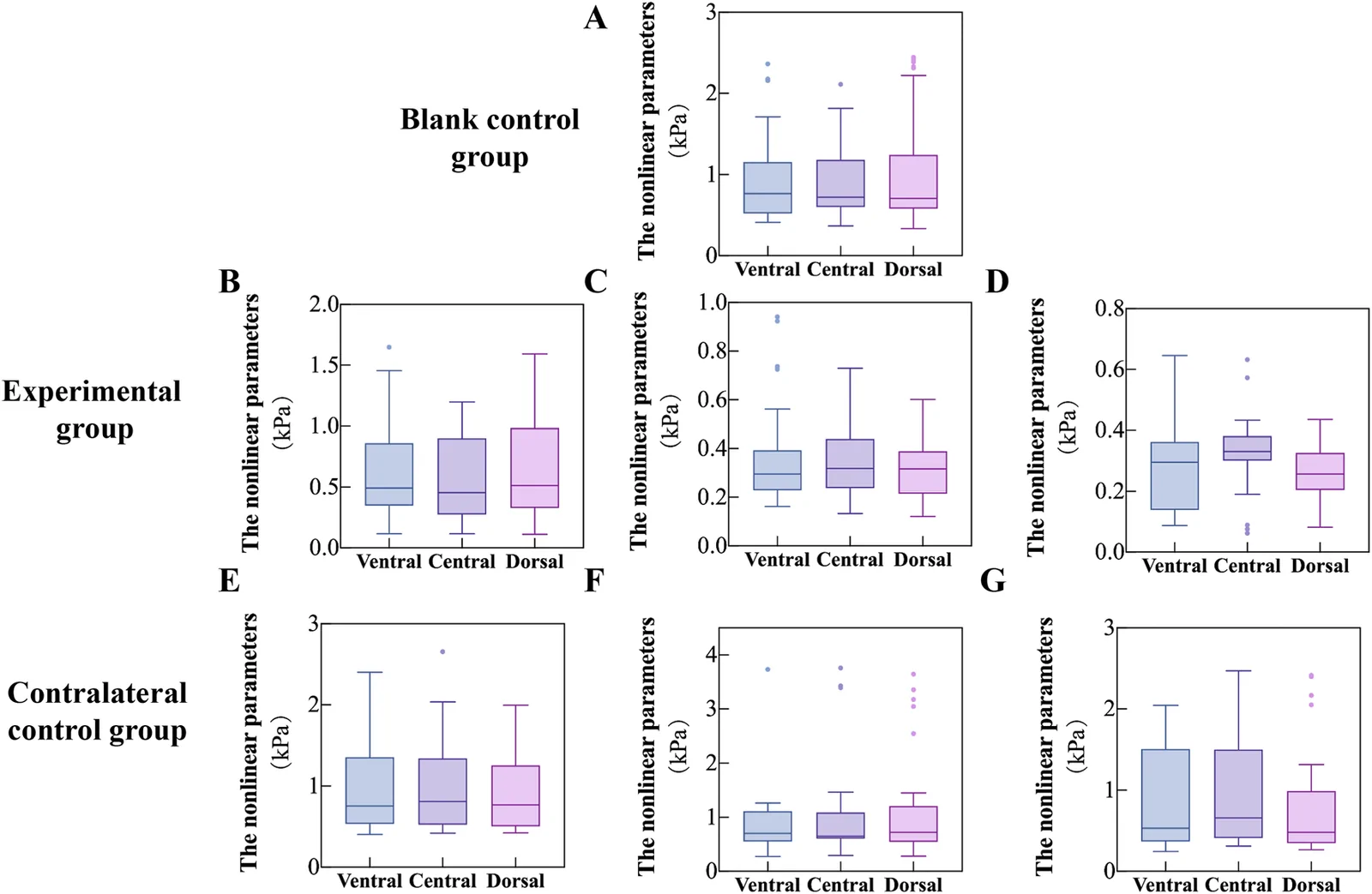
The nonlinear parameter *C*_10_ in the LC across the ventral, central and dorsal regions of each group. **(A)** The blank control group (n = 10), **(B–D)** represent the 4W, 8W, and 12W high IOP experimental groups (n = 6), and **(E–G)** represent the 4W, 8W, and 12W high IOP contralateral control groups (n = 6). No significant differences in *C*_10_ were observed between the ventral, central, and dorsal regions in all groups.

To investigate the variation of the nonlinear parameter *C*_10_ in the LC across different regions, we analyzed parameters in the ventral, central, and dorsal regions of each group (Figure 3). Statistical analysis revealed no significant differences in *C*_10_ between the regions across all groups. Therefore, when investigating the changes in the nonlinear parameter *C*_10_ of the LC at different durations of elevated IOP, regional variations were not considered.

**FIGURE 4:**
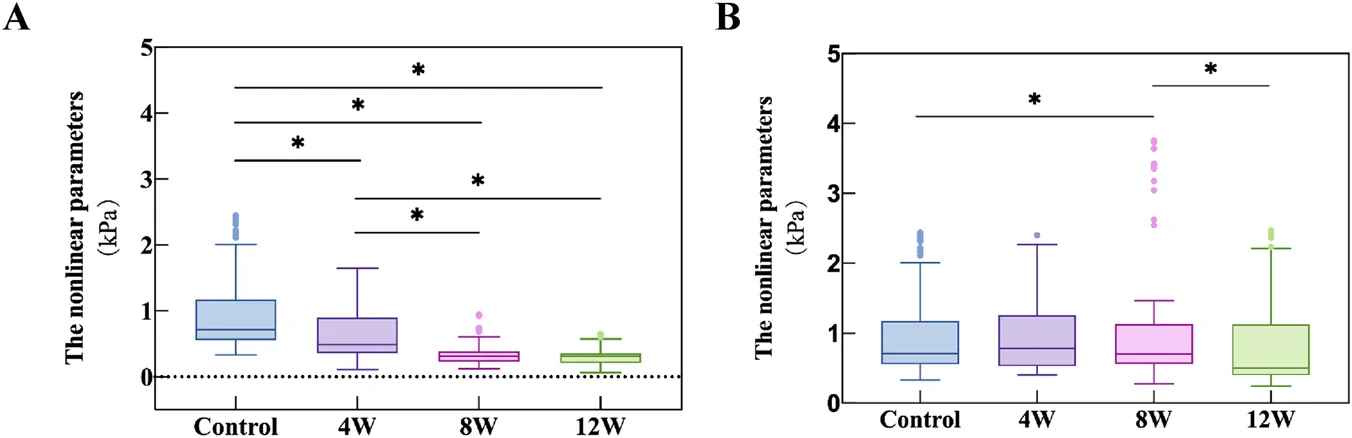
The nonlinear parameter *C*_10_ in the LC at different time points of elevated IOP. **(A)** *C*_10_ values in the blank control group (n = 10) and the high-IOP experimental eyes at 4, 8, and 12 weeks (n = 6 per group). **(B)** *C*_10_ values in the blank control group (n = 10) and the contralateral control eyes at 4, 8, and 12 weeks (n = 6 per group). With sustained high IOP, the *C*_10_ of the LC in the experimental group showed a significant decreasing trend, while the *C*_10_ in the contralateral control group also showed a slow decrease. *Indicates significant differences between groups, with statistical significance based on LSD *post hoc* test results, *P < 0.05.

We performed statistical analysis on the C_10_ values obtained from three different regions in indentation experiments combined together. The nonlinear parameter *C*_10_ shows a significant decreasing trend in *C*_10_ with sustained elevated IOP (Figure 4A). Significant differences in the nonlinear parameter C_10_ were observed between the blank control group and all high IOP experimental groups (*P* < 0.05). Significant differences were also found when the 4W experimental group was compared separately with each of the 8W and 12W experimental groups (*P* < 0.05). Compared with the blank control group, the median *C*_10_ values in the experimental eyes decreased by 31.4%, 56.2%, and 56.5% at 4, 8, and 12 weeks, respectively. In the comparison of the nonlinear parameter C_10_ of the contralateral control eyes in the blank control group and the contralateral control groups (Figure 4B), a gradual decreasing trend was observed after 8 weeks of sustained elevated IOP. Significant differences were observed between the blank control group and the 8W contralateral control group (*P* < 0.05), and between the 8W contralateral control group and the 12W contralateral control group (*P* < 0.05).

Spearman correlation analysis was further performed to calculate the correlation between the nonlinear parameter *C*_10_ of the LC and the duration of elevated IOP in both the high IOP experimental group and the contralateral control group. In the experimental group, *C*_10_ was strongly and negatively correlated with IOP duration (r = −0.684,*P* < 0.05). In contrast, a weaker but still significant negative correlation was observed in the contralateral control group (r = −0.134,*P* < 0.05).

The above experimental results indicate that the sustained effect of high IOP significantly impacts the nonlinear mechanical properties of the LC, with a significant decrease in the nonlinear parameters. This suggests that the LC may undergo structural remodeling, weakening its mechanical support and protective function for RGC axons.

**FIGURE 5:**
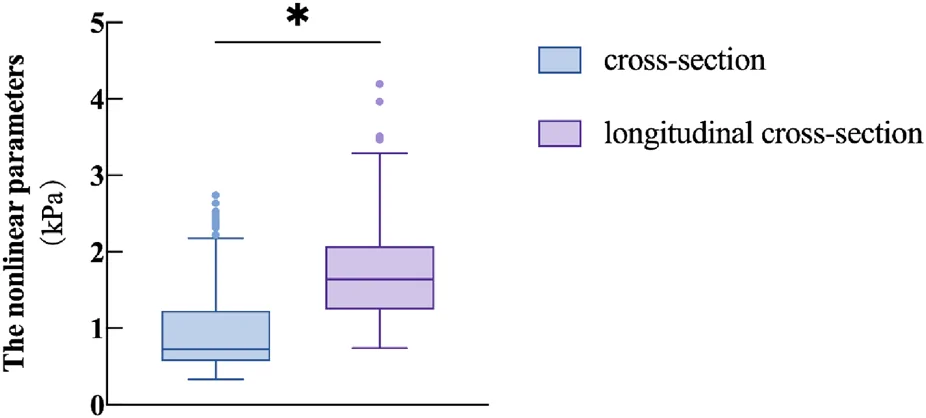
The nonlinear parameter *C*_10_ in the LC of the blank control group for longitudinal and cross-sectional slices (n = 10). *Indicates significant differences between groups, *P < 0.05.

To investigate the mechanical anisotropy of the LC, we prepared longitudinal and cross-sectional frozen sections of the LC from the blank control group. The nonlinear parameter *C*_10_ of the LC in the longitudinal sections of the right eyes from blank control group rats was measured and compared with the *C*_10_ in the cross-sectional slices of the left eyes (Figure 5). The nonlinear parameter *C*_10_ in the longitudinal sections is greater than that in the cross-sectional slices, and the difference is statistically significant (P < 0.05).

### 3.3 Viscoelastic properties of LC

**FIGURE 6:**
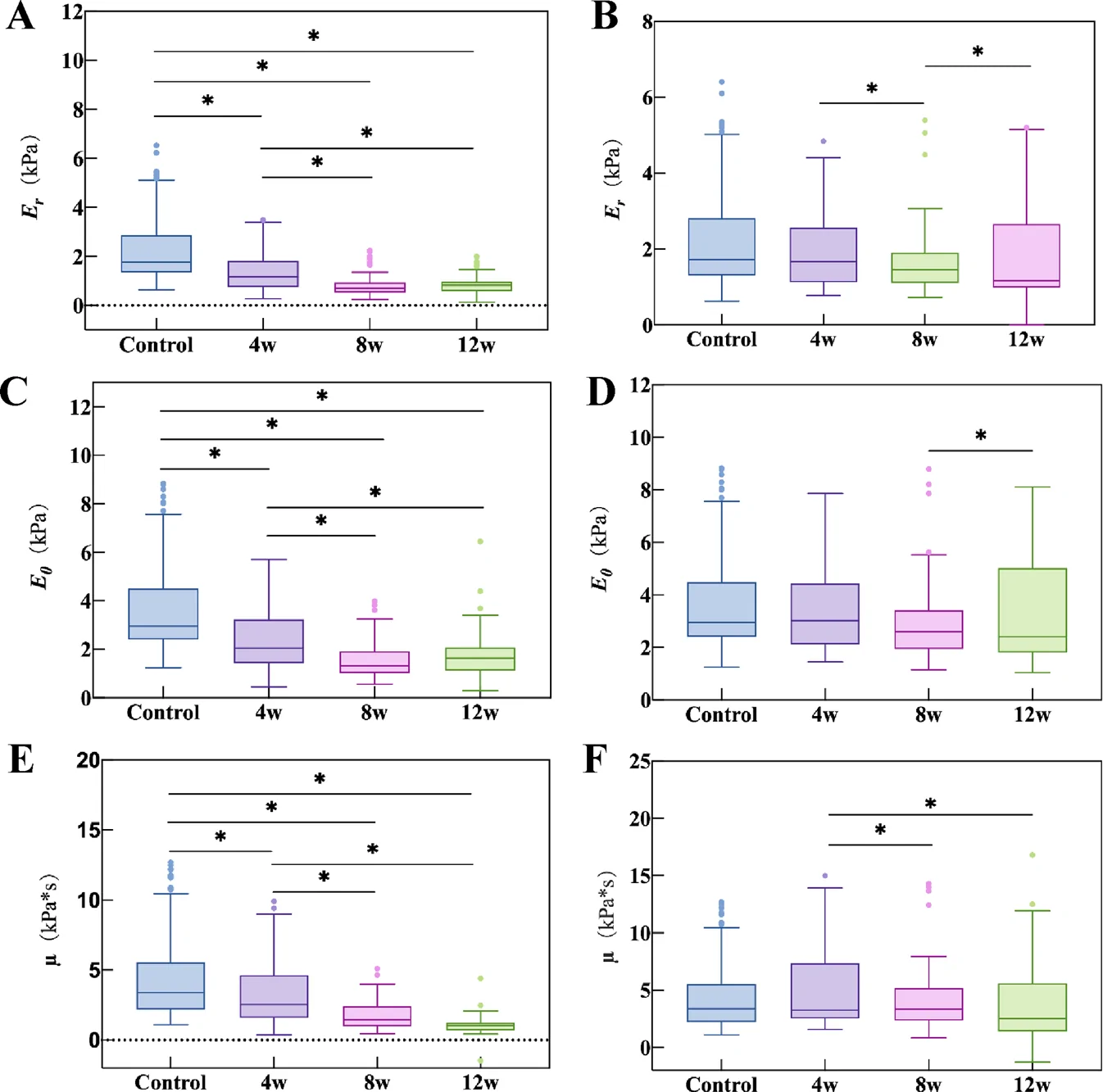
Three viscoelastic parameters of the LC: relaxation modulus $E_{r}$ , instantaneous modulus ${\;E}_{0}$ , and apparent viscosity *μ* under sustained high IOP. **(A,B)** represent the changes in *E_r_* for the high IOP experimental group (n = 6) and the contralateral control group (n = 6), respectively. **(C,D)** Show the instantaneous modulus $E_{0}$ of the LC at different time points of high IOP. (C) and (D) represent the changes in ${\;E}_{0}$ for the high IOP experimental group (n = 6) and the contralateral control group (n = 6), respectively. **(E,F)** Show the apparent viscosity *μ* at different time points of high IOP. **(E,F)** represent the changes in *μ* for the high IOP experimental group (n = 6) and the contralateral control group (n = 6), respectively. *Indicates significant differences between groups, with statistical significance based on LSD *post hoc* test results, *P < 0.05.

To evaluate the effects of sustained elevated IOP on the viscoelastic behavior of LC, we analyze the variations of three viscoelastic parameters (relaxation modulus $E_{r}$ , instantaneous modulus $E_{0}$ , and apparent viscosity *μ*) with respect to the duration of chronic elevated IOP. In the experimental groups, the relaxation modulus $E_{r}$ (Figure 6A) and the instantaneous modulus $E_{0}$ (Figure 6C) decreased progressively with the duration of high IOP. All experimental groups at 4, 8, and 12 weeks showed significantly lower $E_{r}$ and $E_{0}$ values than the blank control group, and additional reductions were detected between the different time points (*P* < 0.05). Compared with the blank control group, the median $E_{r}$ , $E_{0}$ and *μ* values in the experimental eyes decreased by 33.7%, 30.6%, and 25.2% at 4 weeks; by 60.1%, 55.1%, and 57.0% at 8 weeks; and by 53.1%, 44.1%, and 70.0% at 12 weeks, respectively. The apparent viscosity *μ* (Figure 6E) also tended to decline with prolonged IOP elevation, with the 12-week group exhibiting significantly lower *μ* compared with the blank control and 4-week groups (*P* < 0.05). In the contralateral control groups (Figures 6B,D,F), the viscoelastic parameters of the LC also showed time-dependent changes. Significant differences in the relaxation modulus $E_{r}$ were observed between the 4W and 8W contralateral control groups, as well as between the 8W and 12W contralateral control groups (*P* < 0.05). The instantaneous modulus $E_{0}$ showed a significant difference only between the 8W contralateral control group and 12W contralateral control group (*P* < 0.05). The apparent viscosity *μ* was significantly higher at 4 weeks than at 8 and 12 weeks (*P* < 0.05), whereas no significant differences were observed between the blank control group and any of the contralateral control groups or between the 8- and 12-week groups.

Further Spearman correlation analysis was performed to calculate the correlation between the three viscoelastic parameters $E_{r}$ , $E_{0}$ and *μ* of the LC and the duration of elevated IOP in both the experimental group and the contralateral control group (Table 1). The three viscoelastic parameters in the high IOP experimental group showed a strong negative correlation with the duration of high IOP, which was statistically significant (*P* < 0.05). In the contralateral control group, both *E_r_* and *E*_0_ showed significant negative correlations with the duration of high IOP (both *P* < 0.05).

**TABLE 1:** Correlation analysis results between the viscoelastic parameters of the LC and the duration of elevated IOP.

Group	Parameter	*r*	P
Experimental group	$E_{r}$	−0.652	*
	$E_{0}$	−0.582	*
	μ	−0.640	*
Contralateral control group	$E_{r}$	−0.154	*
	$E_{0}$	−0.132	*
	μ	−0.069	​

*Indicates that the Spearman correlation coefficient r is statistically significant, *P < 0.05.

## 4 Discussion

In this study, a rat model of chronic high IOP was established by cauterization of the episcleral veins combined with subconjunctival injection of 5-FU. Indentation experiments were performed on transverse sections of LC region tissues. By fitting the loading segments and stress-relaxation segments of indentation curves using different constitutive formulations, the nonlinear and viscoelastic mechanical parameters of LC were obtained. Then, the time-dependent changes in the nonlinear and viscoelastic mechanical properties of LC under sustained elevated IOP were systematically investigated. In addition, nonlinear mechanical properties of longitudinal sections of LC were measured using the same approach and compared with those obtained from transverse sections to evaluate the anisotropy of the mechanical behavior of LC. These results demonstrated that, similar to other biological tissues (Abass et al., 2019; Hatami-Marbini and Pachenari, 2020; Joyce, Rochev, and Rahmani, 2019; Remache et al., 2018), LC tissues in rats exhibited pronounced nonlinear stress–strain behavior with increasing indentation depth. Moreover, consistent with findings in porcine (Safa, Read, and Ethier, 2021) and rat (Boazak et al., 2019) LC tissues, the rat LC exhibited pronounced stress-relaxation behavior indicating significant viscoelastic behavior. Furthermore, comparison between longitudinal and transverse sections suggested an apparent orientation-dependent nonlinear mechanical response in rat LC. Together, these findings highlight the complex, nonlinear, viscoelastic, and direction-dependent indentation of LC and provide important biomechanical insights into optic nerve injury under chronic elevated IOP.

In the blank control group, nonlinear and viscoelastic parameters obtained from indentation of rat LC were broadly consistent with previously reported ONH mechanical properties. The nonlinear stiffness parameters C_10_ were in the low-kilopascal range, which is comparable in magnitude to the compressive moduli reported for rat and porcine ONH in unconfined compression tests (Boazak et al., 2019) and lies within the same order as the matrix moduli predicted for porcine ONH by viscoelastic mixture modeling studies (Safa, Read, and Ethier, 2021). In contrast, our values are markedly lower than the tensile moduli measured for human LC strips (Spoerl, Boehm, and Pillunat, 2005) and the effective LC stiffness estimated *in vivo* from OCT-based virtual fields methods in humans (Zhang et al., 2017), which typically fall in the tens-of-kilopascal range. These discrepancies are expected and likely arise from differences in species, age and collagen architecture, the tissue region considered, and, importantly, the loading modality. With respect to time-dependent behavior, the stress-relaxation response observed in the LC tissues from the blank control group was consistent with the strong rate-dependent compressive response (Boazak et al., 2019) and the viscoelastic relaxation behavior of porcine ONH described by Safa et al. These findings indicate that both the magnitude and qualitative characteristics of our baseline nonlinear and viscoelastic parameters lie within a physiologically reasonable range.

The statistical analysis of differences among multiple groups demonstrated that, with prolonged exposure to elevated IOP, both the nonlinear mechanical parameters and the viscoelastic parameters of LC in the experimental groups decreased significantly. Spearman correlation analysis further revealed that these nonlinear and viscoelastic parameters were significantly and negatively correlated with the duration of IOP elevation. Although the median IOP values of the 4-week, 8-week, and 12-week experimental eyes were within a relatively close range, the duration of sustained IOP elevation was different among these groups. With prolonged duration of high IOP, the microstructure of LC was remodelled, with a decrease in the LC elastic modulus and substantial loss of RGC axons (Roberts et al., 2009; Zhang et al., 2022; Ma et al., 2025; Guan et al., 2022; Strickland et al., 2022). Therefore, the decline in non-linear properties parameters and viscoelastic parameters is mainly due to the long-term cumulative effects of elevated IOP. This sustained high IOP progressively compromises the mechanical supporting structure of the LC, ultimately leading to optic nerve injury. Notably, although different fitting models were applied to distinct segments of the indentation curves (i.e., the loading segment for nonlinear behavior and the stress-relaxation segment for viscoelastic behavior), the temporal evolution of the nonlinear and viscoelastic parameters in the experimental groups exhibited a high degree of consistency. Specifically, both sets of parameters showed the most rapid decrease within the first 4 weeks of elevated IOP and during the period from 4 to 8 weeks, whereas no significant differences were observed between the 8-week and 12-week time points. These results suggest that the primary period during which the mechanical supporting structure of the LC is damaged occurs within the first 8 weeks of elevated IOP, after which the LC structure may reach a relatively stabilized state. We further analyzed the relative deviations of the nonlinear parameters in the blank control group and the 4-week, 8-week, and 12-week ocular hypertension groups, which were 57.6%, 63.0%, 51.8%, and 41.2%, respectively.

In the contralateral control groups, the nonlinear and viscoelastic parameters of LC also exhibited significant differences at certain time points. Spearman correlation analysis showed that the nonlinear parameters, as well as the relaxation and instantaneous modulus, were weakly but significantly negatively correlated with the duration of elevated IOP. These findings suggest that prolonged elevated IOP in one eye may exert secondary effects on the contralateral eye. Previous studies have demonstrated that elevated IOP induces upregulation of GFAP and MHC-II, along with microglial activation, in the retina contralateral to the eye with experimental glaucoma in mice (Gallego et al., 2012). For different durations of high IOP, Young’s modulus of the glial LC in the contralateral eye showed no significant difference compared with the blank control group (Ma et al., 2025). The nonlinear and viscoelastic parameters of LC were also affected by other factors, including age-related changes during the experimental period, systemic responses associated with the animal model, repeated anesthesia and handling, repeated IOP measurements, etc. Therefore, the results from the contralateral eye require further validation.

Under *in vivo* physiological conditions, tissues in LC are subjected not only to IOP acting perpendicular to the posterior pole of the eye, but also to additional mechanical loads such as radial compression and shear forces exerted by surrounding tissues (Grytz, Meschke, and Jonas, 2011; Coudrillier et al., 2016). Therefore, the mechanical response along different anatomical orientations of the LC should not be neglected. Comparison between different section orientations showed that the nonlinear parameters measured from longitudinal sections were significantly greater than those from transverse sections (*P* < 0.05), which is likely related to microstructural differences along different anatomical directions within the LC (Pazos et al., 2015; Grytz, Meschke, and Jonas, 2011; Morrison et al., 1995). However, this result should be interpreted as an apparent anisotropic response inferred from indentation measurements on differently oriented tissue sections, rather than as a full identification of the three-dimensional anisotropic constitutive properties of the LC. Quantitative imaging studies have demonstrated a pronounced directional distribution of collagen fibers in the ONH related tissues (Pijanka et al., 2012; Voorhees et al., 2018). Moreover, investigations of the human LC have revealed a coupling between the collagen network microstructure and pressure-induced strain, indicating that microstructural anisotropy significantly influences tissue deformation and stress distribution under loading (Ling et al., 2019). In addition, inflation experiments have shown that the human LC exhibits region-specific deformation responses under elevated pressure, further emphasizing the importance of considering directional and regional differences when evaluating its mechanical behavior (Midgett et al., 2020; Kwok et al., 2023).

In this study, we assumed that LC was locally approximated as a homogeneous, nonlinear, and nearly incompressible material. In fact, LC is highly heterogeneous, porous, and direction-dependent. Its microstructure comprises collagenous beams, vascular spaces, glial tissue, and unmyelinated retinal ganglion cell axons, which may all be included to varying degrees within the local indentation region. Although the collagenous beams are generally considered the major load-bearing framework of the LC, non-collagenous components and vascular spaces may also influence the local indentation response through their spatial distribution, hydration state, and interaction with the surrounding collagenous matrix. Our research found that the mechanical properties of glial LC and RGC axons were different (Liu et al., 2022; Ma et al., 2025) using AFM. Previous studies have reported that the mean pore diameter of the human lamina cribrosa is approximately 24.2 μm (Wang et al., 2014). Given that the rat eye is considerably smaller than its human counterpart (Loiseau et al., 2023), when a spherical probe with a radius of 29.5 μm was employed for indentation, the resulting contact area inevitably encompassed the glial lamina cribrosa, retinal ganglion cell (RGC) axons, and other surrounding tissue components. To a certain extent, treating this composite region as a homogeneous material is appropriate. Nevertheless, this inherent structural heterogeneity—including glial LC, RGC axons, blood vessels, and collagenous and non-collagenous tissue structures, among others—may partly account for the observed variability in the measured mechanical parameters, particularly in the control and early-stage ocular hypertension groups (Figures 4A,B). Future studies combining indentation testing with local structural imaging or histology-guided analysis are needed to further clarify the relative contributions of different LC components to its load-bearing behavior.

The nonlinear mechanical and stress relaxation properties were obtained from *ex vivo* tests on tissue sections. *In vivo* biomechanical assessment of the lamina cribrosa (LC) has been explored using optical coherence tomography (OCT)-based approaches combined with inverse mechanical analysis (Sigal et al., 2014; Zhang et al., 2017; Zhang et al., 2020). Furthermore, although techniques like acoustic radiation force optical coherence elastography (ARF-OCE) have mapped elasticity variations in the optic nerve head under different pressures, they lack the resolution to specifically isolate the LC from surrounding tissues (Shi et al., 2023). Therefore, *ex vivo* indentation testing on frozen tissue sections was adopted in the present study. The section thickness was controlled at 50 μm, whereas the maximum indentation depth was limited to 5 μm, approximately one-tenth of the tissue thickness, thereby minimizing substrate effects on the measured mechanical parameters (Long et al., 2011). In addition, previous studies have suggested that controlled freezing has relatively limited effects on the mechanical properties of soft biological tissues (Suto et al., 2012; Reiter et al., 2022). Therefore, the influence of tissue preparation on the measured LC mechanical properties is considered to be within an acceptable range.

The nonlinear mechanical behavior and stress relaxation properties of the material are closely related to the choice of Poisson’s ratio and the influence of fluid redistribution. AFM-based indentation experiments have shown that when the loading rate outpaces intracellular fluid efflux, cell volume remains nearly constant, justifying the use of an incompressible assumption with Poisson’s ratio fixed at 0.5. Conversely, stress relaxation tests reveal compressibility arising from fluid outflow, with the Poisson’s ratio directly derivable from the pre- and post-relaxation force measurements (Mollaeian et al., 2018). Since LC is a porous load-bearing tissue (Cavuoto et al., 2026; Causin et al., 2014), its time-dependent behavior is influenced by the Poisson’s ratio and the influence of fluid redistribution. Uniformly applying a Poisson’s ratio of 0.5 may introduce certain inaccuracies. Meanwhile, poroelastic modeling studies have shown that LC deformation is closely associated with interstitial fluid flow, tissue permeability, and pressure redistribution within the optic nerve head (Causin et al., 2014; Cavuoto et al., 2026). During indentation, local deformation of the porous LC may generate pressure gradients and fluid redistribution within the tissue, which could contribute to the measured relaxation response. Future studies combining indentation testing with poroelastic or poro-viscoelastic constitutive modeling may provide a more comprehensive understanding of LC mechanical behavior under chronic ocular hypertension.

This study has several limitations that should be considered when interpreting the findings. First, all mechanical tests were performed *ex vivo* on sectioned rat LC tissue, which cannot fully reproduce the *in vivo* loading environment, boundary conditions, or fluid perfusion of the ONH; as a result, the absolute magnitudes and characteristic time scales of the reported parameters may differ from those of the intact LC. Second, the indentation protocol probed local through-thickness compression at small depths, and each section was effectively modeled as a homogeneous, and nearly incompressible material, though the LC is a highly heterogeneous, porous and direction-dependent tissue. More advanced constitutive descriptions might change the quantitative values of the fitted parameters. Third, the apparent anisotropy was evaluated only by comparing longitudinal and cross-sectional sections in a single anatomical region and time point in blank control eyes. Therefore, these results do not represent a full identification of the three-dimensional anisotropic constitutive properties of the LC, nor do they capture regional variability across the ONH. Future studies incorporating three-dimensional microstructural reconstruction and anisotropic constitutive modeling are needed. Fourth, the chronic IOP elevation model used here (episcleral vein cauterization combined with subconjunctival 5-FU) and the discrete observation time points (4, 8, and 12 weeks) in a relatively small cohort may not capture the full spectrum of glaucoma phenotypes or inter-animal variability. Finally, we focused on changes in mechanical parameters and inferred structural remodeling only indirectly; detailed correlations between LC microstructure, mechanical degradation, and functional outcomes such as RGC loss were beyond the scope of this work and should be addressed in future studies.

In summary, this study quantitatively characterized the nonlinear and viscoelastic mechanical properties of rat LC and revealed their evolution under chronic elevated IOP. The results indicate that prolonged high IOP significantly impairs the mechanical support of LC. Moreover, measurable changes in LC mechanical parameters were also observed in the contralateral eye under unilateral high IOP, indicating that IOP-induced optic nerve damage may involve bilateral effects. These findings emphasize the importance of the nonlinear, viscoelastic, and anisotropic mechanical behavior of LC in glaucoma pathogenesis and provide a biomechanical basis for future modeling studies and therapeutic strategies targeting optic nerve protection.

## Data Availability

The original contributions presented in the study are included in the article/Supplementary Material, further inquiries can be directed to the corresponding author.
