# Supplementary material for: Changes in the nonlinear and viscoelastic mechanical properties of the rat lamina cribrosa under chronic high intraocular pressure
Source: Front Bioeng Biotechnol. 2026 Aug 31;14:1875609. doi: 10.3389/fbioe.2026.1875609 (PMC13572659; doi:10.3389/fbioe.2026.1875609)
Supplement: Supplementary file 1 [file Supplementaryfile1.docx]

Supplementary Material

**Supplementary Figure 1.** Experimental workflow for tissue preparation and indentation testing
